# Lectures on the Eruptive Fevers, as Now in the Course of Delivery at St. Thomas’s Hospital, London

**Published:** 1851-10

**Authors:** 


### REVIEWS.

---

ART. III—*Lectures on the Eruptive Fevers*, as now in the course of delivery at St. Thomas's Hospital, London. By GEORGE GREGORY, M. D., Fellow of the Royal College of Physicians of London; Physician to the Small-Pox and Vaccination Hospital at Highgate; Corresponding Member of the National Institute of Washington, etc. First American edition, with numerous additions and amendments by the Author, comprising his latest views: With notes and an appendix, embodying the most recent opinions on exanthematic pathology; and also statistical tables, and colored plates. By H. D. BULKLEY, M. D., Physician of the New York Hospital; Fellow of the New York College of Physicians and Surgeons, etc. etc. p.p. 379, 8vo. New York: S. S. and W. Wood, Publishers. 1851.

These Lectures were delivered and published in London, in 1843, and have been repeated at St. Thomas's Hospital every year since. Of the importance of the subject of which they treat a more forcible illustration could not be given than the fact, that the eruptive fevers together with hooping cough "absorb one ninth of the mortality" of Great Britain and probably of all other countries. The skin is a highly sensitive organ, and exercises a controlling influence over the animal economy. "A man may lose one half of his lungs by slow ulceration, and he may yet live for months, nay, for years. But if one half, or one third, or even one fourth of the skin of the body be destroyed, the system rapidly gives way, and death ensues." The skin is the fourth in the series of important organs, yielding only to those of the encephalon, the chest, and the abdomen.

Dr. Gregory divides his subjects into the greater and lesser Exanthemata; the former embracing Small Pox, Measles, Scarlatina, and Erysipelas; the latter Vaccinia, Varicella, Herpes, Miliaria Lichen, Urticaria, Roseola, and Erythema. By the first class, life is brought seriously into danger; the latter are comparatively harmless. In some years, the mortality from the acute exanthemata rises in Great Britain as high as thirteen per cent., and in others falls as low as six; but it is a curious fact, adverted to by Dr. G., that "the epidemic mortality remains, on an average of years, nearly the same," some other disease coming in to equalize the fluctuations. This law of "*vicarious mortality*" is exemplified in a table, showing that in a series of years, whenever one epidemic diminished another increased—that if small pox prevailed, scarlet fever and measles were less prevalent, but that every year was distinguished by some "master epidemic." In 1838, the mortality from small pox, in England and Wales, was 10,268; from measles, 6,514; from scarlet fever, 5,802. In 1839, from small pox, it was 9,131; from measles 10,937; and from scarlet fever, 10,325. In 1840, it was 10,434 from small pox; 9,326 from measles, and 19,816 from scarlet fever.

Statistics bring out startling conclusions. We are in haste to assume that our remedial and hygienic measures have wrought great changes in the results of disease, and that the average mortality of the species is growing annually less. But alas! these figures teach us a different lesson. As one avenue to death is closed, another opens. Vaccination bade fair, for a time, to blot out small pox from the list of human infirmities; but "the blessings of vaccination are met and counterbalanced by the law of vicarious mortality." It would seem to be a law of nature, using the words of Dr. Gregory, "that the weak plants of a nursery must be weeded out." If weakly children do not fall victims to small pox, they live to fall into the jaws of tyrants scarcely less inexorable. Scarlet fever and measles are both advancing in respect of mortality, and the increase of deaths by whooping cough since this century set in, is quite extraordinary." Statistics prove, as remarked by Dr. Bulkley, that the per centage of deaths under five years of age is rather on the increase than the contrary, in cities at least; or, to speak most favorably, remains about stationary.

Statistics show that this law is of wide application, and holds good of other than exanthematous diseases. The mortality of any given city or country varies, from year to year, much less than is generally believed, and is even much less affected than we are apt to conclude by great epidemics. When these prevail other diseases fall off, and "if men die of cholera, or children of small pox, they are not left to the prey of pneumonia, or of hydrocephalus, of asthma or of croup."

The peculiarities of exanthematous diseases are the following:—the eruption, the universal susceptibility, the non-recurrence, the contagion, the epidemic diffusion. In common with most other diseases, they are generally attended with fever; but this phenomenon is not always present, and so far from being necessary to the natural process of the complaint, violence of initiatory fever is always injurious. It disturbs the regular march of the exanthem, and one of the chief offices of the physician is to subdue it, in order that the eruption may appear, and the disease have a chance of running a safe course.

As, then, there may be eruption without fever, it may be asked, "may there not be the specific fever of an exanthematous poison without eruption?" Can a man go through small pox or measles without exhibiting eruption? An affirmative answer has been given by eminent observers, and a recent epidemic of measles in Paris seems to have created an extensive impression that the constitutional symptoms of the disease may occur without the eruption; but the "pathology" is regarded by Dr. Gregory as "very questionable."

The law of universal susceptibility is an interesting character of eruptive fevers. One may expect to go through life without experiencing the pain of a great number of complaints, but every one counts on an attack of measles, and only expects to escape small pox by means of vaccination. Willis says, "that the escape of a man living to the ordinary period of human life from small pox and measles, is as rare as the falling into them twice." Some, however, do escape, as some are twice affected, and, what is a still more curious fact, there are some countries in the world in which the exanthemata are still strangers. Small pox, measles, and scarlet fever, according to the testimony of medical travelers, are to this day unknown in Australia and Van Dieman's Land—these countries of strange productions—the land of the marsupialia, of the ornithorynchus, and of black swans, being also impressed with the physical qualities which exclude this branch of "the painful family of death."

"Non-recurrence" is another feature of the exanthemata, but is not a universal law, for instances of second attacks of measles and small pox are but too well established. The law holds good in respect to other disorders than the exanthematous, and though not as true of yellow, typhus, and scarlet fever, as of small pox, the testimony of all observers is that those who have once suffered with these fevers are in slight danger of a second attack. But we cannot agree with Dr. Gregory, that "it belongs in a certain limited degree to fevers of a paludal origin." On the contrary, it has appeared to us that the oftener one has had remittent or intermittent fever, the more prone he is to such attacks. And so far from being true, as he contends, that "in all cases the susceptibility of a disease is more or less exhausted by once undergoing it," we have always supposed that the tendency to certain complaints was increased by each recurrence. Pneumonia, for example, we had thought was more apt to occur for having once been excited, just as the joint once dislocated is more easily displaced ever afterwards.

The last characteristic of the exanthemata but one, is their "contagious origin," and that they operate upon the healthy body without the aid of predisposing causes. A man in the most perfect health contracts measles or small pox as readily as one enfeebled by previous disease, and such a state of his system is the fittest by far for insuring the success of vaccination. The poison of these diseases seems to originate in the blood, and to be communicable directly by the blood, without the agency of secretion. Glanders is communicated to a healthy horse by the injection of the blood of a glandered horse into his veins; and the same has lately been proved to be true of measles by experiments in Austria, in so many cases that no ground is left for skepticism on the subject. But they are communicable by other modes.

"All miasms of animal origin are capable of attaching themselves to fomites, and (provided they be excluded from the air) of retaining their communicating property for a considerable length of time. This great law of nature is the foundation of that important practical measure —quarantine. It is a law of universal application. Tinea capitis spreads by means of hats, combs and brushes; Egyptian ophthalmia by towels and sponges; small pox and typhus by clothes and bedding; plague by personal apparel and old rags. Some would persuade us that merchandise, which, *ex necessitate rei*, could never have been near the chambers of the sick, or handled by others than by men in health, may also communicate contagion; but I believe this doctrine to be opposed to every principle in sound pathology."

All the evidence respecting the propagation of cholera appears to us to be tending to the same point. The facts going to show that it is *portable* are multiplying every day. It does not always go abroad with those who have been in the midst of it; it is not invariably transported by those who have been exposed to the infection. Far otherwise; in a large majority of cases, those who have breathed the pestilential air, and been with the sick and dying, fail to disseminate it; but still when disseminated, it seems to be through the same agency which diffuses the poison of the exanthemata. The striking difference between cholera and these contagious diseases is, that they require no favoring influences to propagate them, while it only spreads when the infection is transported to a favorable soil. Cholera must have adjuvants, and hence so often fails to spread where the poison exists. Small pox and measles are wholly independent of climate, soil, or season.

The other character of the exanthemata mentioned by Dr. G. is their occurrence as epidemics. Small pox has been repeatedly epidemic in this country, during the last few years, and in that form is more fatal than when only occasional cases are occurring. Dr. Bulkley remarks that,

"Epidemic diseases seem to be more fatal among the uncivilized than the civilized. In an epidemic of Rubeola among the Crees (a tribe of North American Indians) in the summer of 1846, as reported by Dr. Smellie (*Monthly Journal of Medical Science*, December, 1846), in 145 cases treated in their camp, 40 were fatal.

"Small pox has sometimes swept off an entire tribe of Indians, as was the case with the Mandans (another North American tribe), and has been raging with great fatality among some other tribes of these Indians during the past and present year.

"These races ascribe epidemics to spells exerted by their enemies, or regard them as direct visitations of the Great Spirit, and abandon all hope of recovery as soon as attacked.

"They seem also to differ in the degree of their mortality among blacks, small pox, measles, and hooping cough, being more fatal to them than to whites, while scarlet fever would seem to be more fatal to whites.

"In Charleston, (S. C.), the mortality by small pox during the period from 1822 to 1848 inclusive, was 49 among the white population and 154 among the blacks. Within the same period, 289 whites and 163 blacks died with scarlet fever, and 45 whites and 97 blacks died with measles; and 100 whites and 257 blacks with hooping cough,—the population of the whites being 12,828, and of the blacks 17,461, in 1830; and 13,000 whites, to 16,-200 blacks, in 1840."

Exanthematic debility, Dr. Gregory remarks, is apt to be mistaken, and to lead to bad practice. The debility most generally depends upon secondary fever, and is to be treated, not by beef-tea, wine, bark and tonics, but like other fevers, by purgatives, diuretics and low diet.

Dr. Gregory thus concludes his general remarks on the exanthemata:

"When all is done, you will not fail to remark how small a proportion the strictly therapeutical and practical parts of the course bear to the descriptive and pathological portions; it will often remind you of Falstaff's *ha'porth* of bread to his two gallons of sack. Remember, however, that in the exact proportion in which we improve the two latter, we diminish not the importance, but the extent, of the former. In the early periods of medicine, when descriptions of disease were imperfect, and pathology was in its infancy, and statistics were unknown, physicians arrogated to themselves a power of controlling, by drugs, the course of diseases (and especially of exanthematic diseases), which we now know to be wholly unwarranted. Pages and chapters were devoted to objects quite unattainable; presenting, indeed, an imposing, but a vain parade of learning. In this respect we have improved upon our predecessors. We are not ashamed to acknowledge that many diseases *must* run their stated course, and that others will run their course, in defiance of all the efforts of medical skill. In the management of the exanthemata, be satisfied with steering the ship. Do not attempt to quell the storm. Trophilus, an ancient Greek physician, being asked who was the most perfect physician, replied, "He who knows best how to distinguish that which can from that which cannot be done." "

The three following lectures are devoted to small pox, the first opening with a brief sketch of the early history of the disease. Dr. Gregory does not believe from all he has read on the subject, that small pox was known to the Greeks and Romans, and it is certainly a pretty strong argument in favor of this opinion, that in the writings of Alexander Trallianus, who attempted to give a description of the whole circle of medical science as it existed in the sixth century, no allusion is made to any complaint exhibiting the character of variola. Rhazes described it with great clearness in the year 910, and he is the first author who mentions it, though there can be no doubt that the disorder had been known for several centuries before it was described. Originating in the east, in the countries bordering on the Red Sea, it travelled to the west, and reached England about the close of the ninth century. "The word variola," says our author, "is to be found in several Latin manuscripts preserved in the British Museum, of date decidedly prior to 900." It reached our continent about 1527—according to Humboldt, in 1520—having been introduced by a negro slave into Mexico, whence it spread in all directions. In 1633, the Indians of Massachusetts were invaded by the fatal disease, which swept them away by thousands. It unfortunately happened that about this period, the hot or sweating system in the treatment of fevers had attained its acme, and as the method was deemed especially applicable to small pox, it is but too evident that the appliances of medical art conspired with the disease to swell its mortality. We realize with some difficulty at this day the monstrous absurdity of that system, a specimen of which we present as a curiosity. It is from Diembroeck, and runs thus as quoted by Dr. Gregory:

"Keep the patient in a chamber close shut. If it be winter, let the air be corrected by large fires. Take care that no cold gets to the patient's bed. Cover him over with blankets. Red blankets have always been preferred —not that the color is material—but because, in the times of our ancestors, all the best, thickest, and warmest blankets, were dyed red. Never shift the patient's linen till after the fourteenth day, for fear of striking in the pock, to the irrecoverable ruin of the patient. Far better is it to let the patient bear with the stench, than to let him change his linen, and thus be the cause of his own death. Nevertheless, if a change be absolutely necessary, be sure that he puts on the foul linen that he put off before he fell sick, and, above all things, take care that this supply of semi-clean linen be well warmed. Sudorific expulsives are, in the meantime, to be given plentifully, such as treacle, pearls, and saffron."

It may be questioned whether the brilliant discovery of vaccination by Jenner, some years later, was a greater benefaction to the race than the cooling plan of practice introduced and established by Sydenham. But we dismiss the history of small pox with one more quotation, simply remarking that the sketch of our author though condensed is yet a pleasing one:

"In 1798, Dr. Jenner announced the discovery of vaccination. On the 5th of May, 1808, the inoculation of out-patients was discontinued at the Small Pox Hospital. On the 20th of June, 1822, inoculation was discontinued to in-patients. On the 23d July, 1840, the practice of inoculation, the introduction of which has conferred immortality on the name of Lady Mary W. Montague, which had been sanctioned by the College of Physicians, which had saved the lives of many kings, queens, and princes, and of thousands of their subjects, during the greater part of the preceding century, was declared illegal by the English parliament, and all offenders were to be sent to prison, with a good chance of the treadmill. It is even provided that an attempt to produce small pox by inoculation, even though unsuccessful (including, of course, the testing of vaccinated subjects at all ages), is an offence at law! Such are the reverses of fortune to which all sublunary things are doomed."

Nor shall we stop to give any account of the character of the eruptive fever, the pustule, the maturative stage, or the various complications of small pox, all of which are treated at length and with clearness, but proceed to condense some of the statistics of the disease.

The statistics of small pox have attracted attention from the earliest periods, and the old English bills of mortality, it is said, can be trusted to more in it and plague, than in any other disorder. They "give 199,-665 as the total amount of deaths by small pox in London, during the last century, of whom 97,546 perished in the first half, and 102,119 in the second half." Anterior to the discovery of vaccination, "the proportion of the mortality by small pox to the total mortality was as 8 to 100 in London," and there is no reason to doubt that it was less in other parts of the kingdom.

Children, in all ages, have been the greatest sufferers from small pox. Haygarth computed, in 1795, that it carried off half the children that died, at Chester, below ten years of age; and from reports lately made to Parliament it appears that, "out of every nine persons who now die of small pox in England, seven are below the age of five years." Of 758 persons who died of this disease in New York, from the year 1840 to 1844, inclusive, 411 were under five, and 486 under ten years of age.

Notwithstanding all that vaccination has done, it was found by the registrar-general that there were still only four diseases which, in England, stood before small pox, "with reference to the actual amount of mortality," namely, consumption, convulsions, typhus fever and pneumonia. Taking the world over, Dr. Gregory estimates the average mortality by small pox "at one in six of those attacked," and this in spite of all the improvements in regimen and medical treatment.

It is an interesting question how far the character of the disease has been modified by vaccination, and tables illustrative of this are given by our author, from one of which we gather that "among 396 unprotected cases, there were only 23 which were mild in their aspect; while out of 298 vaccinated subjects, there were no less than 134 which presented, from the onset, favorable appearances, independent of 66, which displayed modification during the maturative stage." Other tables show, that of 6,222 persons admitted into the small pox hospitals of England, in 21 years, 2,530 had been vaccinated; and that while but 164 of this number died the total number of deaths was 1,530, or 1,366 among the unprotected. The result of the whole is, that "small pox in the unvaccinated is five times more fatal than it is in those who have previously undergone vaccination." But these statistics, while they prove that the danger of the disease is greatly mitigated by vaccination, establish the fact, that the idea of banishing it from the earth by vaccine inoculation is indeed, as our author expresses it, "vain and illusory."

Dr. Gregory holds to the doctrine, that the protective power of cow pox is complete for a given period but diminishes later in life. He says:

"It is a matter of general notoriety that small pox is very seldom taken by vaccinated children who are under the age of eight years. In the course of a long experience at the Small Pox Hospital, I have never seen more than three or four instances of such an occurrence."

He does not however believe, that this diminution takes place in all cases, nor does he pretend to give the proportion of such cases to those in which the protection is complete.

"But though this be impossible, there seems no reason why we should not attempt to ascertain the laws which affect and limit that power of resistance to the variolous virus which cow pox displays in so many instances, and so remarkably in infantile life. I have mentioned puberty as a disturbing cause. I have no doubt that others exist, of equal, perhaps of superior efficacy. Among them may be mentioned change of climate, which appears to have a very marked influence, sufficient to induce us to recommend the re-vaccination of all young people going to or returning from India. A severe fever, in like manner, may so alter and modify the general mass of fluids as to open a door to the reception of the variolous effluvium. Importance should be attached also to the epidemic constitution of the season. It is certain that persons who under common circumstances have, through the agency of the cow pox, resisted the variolous miasm, succumb to it under epidemic visitation."

It becomes, therefore, a question of the most serious import, whether re-vaccination will renew the immunity from small pox. The physicians of Germany have been strenuous advocates for the practice, and assuredly they are not without experience to sustain them in it. Thus, they report 14,384 cases of re-vaccination among soldiers without a case of variola in a space of five years; and 26,864 civilians re-vaccinated, with only three cases of small pox in the same period. During an epidemic small pox in Copenhagen extending through several years, they state that "not a single instance of variolous or varioloid disease was observed among any who had been re-vaccinated." Many similar instances might be cited, but it is unnecessary. The operation, to say the least of it, is almost always a harmless one, and there can be no objection to its performance.

Whether small pox ever originates spontaneously, or is not dependent in every case upon a specific virus, is a question that cannot yet be said to be fully settled. That the poison sprung originally from "some fortuitous combination of common causes," as Boerhaave held—that, in other words, it began without *contagion*, there can be no doubt; and if it commenced thus, there is certainly a possibility that it may in this manner occasionally spring up again, but the contingency is not a probable one, and we incline to the theory of *an invariable origin from contagion*. But while adopting this view, we are not to lose sight of the facts which connect it with other epidemic maladies, for that it partakes of this character is as indisputable as that it is contagious. In the last hundred years it has prevailed six times in London in a form eminently epidemic, and from the year 1649 to 1764, according to Webster, it has appeared epidemically nine times in Boston. More than once it has followed cholera as an epidemic in some of the large cities of our country, and prevailed in this place, to a considerable extent last autumn and winter after the disappearance of that disease.

We may advert to another question, rather curious than practical, before dismissing this subject; namely, the identity of small pox and cow pox. It is known to have been a favorite notion of Jenner, that cow pox is the parent of variola—that small pox is vaccinia converted into a malignant form by time and unfavorable circumstances; and this has been the generally received opinion on the subject. But the facts upon the whole, appear to us to be opposed to it, and we agree with our author that, instead of being identical, cow pox and small pox "are antagonist affections, striving to gain the mastery of the human frame, and each under different circumstances and at different times, proving successful;"—that, as expressed by M. Bousquet, "there is no identity of nature, but a reciprocity of action." At the same time, we are aware that there are those of high authority who consider that the question is settled in favor of the identity of the two affections, and who hold that this fact supplies a powerful argument in favor of vaccination.

We have seen what is the actual amount of the protection which vaccination affords; we have seen that it is very great—that small pox in the vaccinated is five times less fatal than in those who are unprotected; but we have also seen, as is now universally admitted, that the loathsome disease will come, in very many cases, in spite of vaccination. With the protection now afforded by the prophylaxis, Dr. Gregory thinks we "must rest satisfied." He does not believe the result will be affected "by any additional precautions on the part of vaccinators," or any "alteration in the kind of lymph employed." And this leads him to propound the following serious question—whether we might not find in adult inoculation a better mode of testing and improving the security of the vaccinated? Upon which he goes on to remark:

"Small pox, taken casually after vaccination, proves fatal, as we have seen, at the rate of seven per cent Inoculated small pox proved fatal, in former times, at the rate of only one-fifth per cent., or one in five hundred. As regards the extension of small pox from the practice of inoculation, no real danger need be apprehended. The experience of ten years in England has amply demonstrated that the diffusion of small pox is wholly independent of such artificial propagation. Small pox has been as general in England since inoculation was abolished as it was previously. But it is as yet little known, that small pox may be received into and pass through the system without producing either fever or eruption. I inoculated three of my own children at the respective ages of twelve, thirteen, and fourteen, after successful vaccination in infancy, and the result was as follows:—In two, local affection without any fever or eruption. In the third case, local affection without fever, but with papular eruption on the seventh day, not advancing to vesicles. I firmly believe that these children are now, and will remain through life, unsusceptible of small pox. The advantages derivable from such a course of procedure are these:—If the child's constitution be under the full vaccine influence, no effect will follow. If the vaccine influence be subsiding or altogether lost, then the small pox will be taken at the period of life (puberty) most favorable for safety, instead of being received (as too often happens under the present system) under circumstances the most unfavorable,—for instance, by mothers at the period of parturition, by young women on the eve of marriage, or by young men at a distance from their friends. The state of the law in England prohibits the practice here; but I hope it will be tried in other countries, where the judgments of medical men are less fettered."

We have dwelt at so much length upon these interesting topics, that we shall be obliged to pass hurriedly over the remaining subjects of these lectures. These are Measles, Scarlatina, Erysipelas, Vesicular Eruptions, and non-contagious efflorescences, to which much valuable matter is added by the editor in the form of an appendix.

It is the prevalent belief among our best medical historians, that measles began to spread through the world about the same time as small pox, and that it arose in the same countries whence the variolous poison had its origin. The miasm, Dr. G. believes, is invariably received by the mode of infection. For a long time it was held by medical men to be identical with the poison of small pox, as scarlatina and measles were regarded as modifications of the same affection until comparatively a late period. Sydenham attacked successfully the former notion, and at the present day no physician believes that the latter complaints originate in a common infection.

Pneumonia is one of the most formidable complications of measles; it occurs both in the progress of the eruption and during its decline. Dr. G. remarks:

"It is a slow, creeping, insidious form of inflammation, which too often throws the practitioner off his guard. No positive complaint is made. The child droops, and appears weak and exhausted. Imagining that the disorder has weakened his patient, the practitioner directs some mild tonic. Meanwhile, pneumonic engorgement (or pneumonia in its first stage) creeps on. The lungs become more and more congested, and at length solidified. A convulsive fit now takes place. Alarm is taken, and leeches are applied. but the mischief is irreparable. Dyspnœa increases. The child becomes drowsy, the feet cold. The pulse sinks. Fluid effusion now takes place from the bronchial membrane. Another and another fit succeeds. Rattles are heard in the throat. The child dies!"

A physician who is on his guard will detect this latent inflammation, and at least not suffer it to proceed to a fatal termination without an effort to save his little patient. But there is an inflammation of a different kind, in which tubercles are developed and small abscesses formed—in which the child becomes emaciated, consumptive, and dies. Dr. Gregory believes, that nine-tenths of the deaths by measles occur in consequence of this subacute form of pneumonia.

Few complaints vary more in intensity than scarlet fever. It is sometimes the mildest, and again the most malignant of diseases. Often the patient passes through it with only the slightest indisposition; in other cases it hurries its victims off with the suddenness of epidemic cholera. It is a fever sometimes typhoid, at others inflammatory, but always to be dreaded when it assumes the form of an epidemic. There can be no question that it is an increasing malady in our country, but happily not yet, like measles, one which all persons may expect to experience sometime in the course of their lives. From a number of observations quoted by Dr. Gregory, it appears that the medium rate of mortality by scarlet fever in Great Britain is six per cent., upon which he remarks: "What a picture may be thence drawn of the actual extent of scarlet fever in years of epidemic prevalence! It shows that in London, in 1839, there were 41,650 cases of this disease; while throughout England and Wales, in 1840, when 19,816 persons died, the total number of seizures must have reached the almost incredible number of 330,266."

In a large proportion of cases, Dr. Gregory believes that scarlatina is the produce of a specific poison; but he admits that it may arise from other causes. He seems not to attach the slightest importance to belladonna as a preventive of this disease, not even so much as alluding to it. His remarks on the treatment of scarlet fever are judicious and valuable, but we must pass them over for want of room; as indeed we are compelled to do the remaining parts of this interesting work. In taking leave of it, we would cordially recommend it to our readers, as the production of a man who has thoroughly investigated the subjects of which it treats. We have risen from the perusal of it with a high appreciation of the author's judgment, learning and ability. Y.

